# The Role of Early Growth Response Family Members 1–4 in Prognostic Value of Breast Cancer

**DOI:** 10.3389/fgene.2021.680132

**Published:** 2021-06-09

**Authors:** Leiyu Hao, Fengru Huang, Xinqian Yu, Bujie Xu, Yan Liu, Yan Zhang, Yichao Zhu

**Affiliations:** ^1^Department of Physiology, Nanjing Medical University, Nanjing, China; ^2^Research Division of Clinical Pharmacology, First Affiliated Hospital of Nanjing Medical University, Nanjing, China; ^3^Department of Gynecology and Obstetrics, Wuxi Maternal and Child Health Hospital Affiliated to Nanjing Medical University, Wuxi, China; ^4^State Key Laboratory of Reproductive Medicine, Nanjing Medical University, Nanjing, China

**Keywords:** EGR, expression profile, prognosis, migration, breast cancer

## Abstract

Early growth response family members (EGRs), EGR1–4, have increasingly attracted attention in multiple cancers. However, the exact expression patterns and prognostic values of EGRs in the progress of breast cancer (BRCA) remain largely unknown. The mRNA expression and prognostic characteristics of EGRs were examined by the Cancer Genome Atlas (TCGA), Oncomine, and Kaplan-Meier plotter. Enrichment analyses were conducted based on protein-protein interaction (PPI) network. The Tumor Immune Estimation Resource (TIMER) database and MethSurv were further explored. The protein expression of EGR1 in BRCA was measured by western blotting and immunohistochemistry. The migration of mammary epithelial cells was determined by Boyden chamber assay. The transcriptional levels of EGR1/2/3 displayed significantly low expression in BRCA compared with that in normal tissues, while EGR4 was shown adverse expression pattern. Survival analysis revealed upregulated EGR1–4 were remarkably associated with favorable relapse-free survival (RFS). A close correlation with specific tumor-infiltrating immune cells (TIICs) and several CpG sites of EGRs were exhibited. Immunohistochemistry assays showed that the protein expression of EGR1 was remarkably downregulated in BRCA compared with that in paracancerous tissues. The migration of MCF10A mammary epithelial cells was increased after the silence of EGR1 by siRNA transfection. This study provides a novel insight to the role of EGRs in the prognostic value of BRCA.

## Background

Breast cancer (BRCA) remains one of the widespread and main fatal malignancies in female diseases worldwide (Nazih and Bard, 2020; Padmanabhan et al., 2020). However, the overall survival (OS) and release-free survival (RFS) of patients with BRCA remain far from satisfaction (Harbeck and Gnant, 2017). Nevertheless, it is difficult for patients with high risk to be diagnosed timely in the early screen system and to be evaluated accurately before postoperative recurrence, owing to lack of reliable and efficient biomarkers (Ronchi et al., 2020). Moreover, personalized treatments are increasingly concerned with the advent of precision medicine (Li and Warner, 2020; Malone et al., 2020). Therefore, the novel potential biomarkers for BRCA treatment need to pay more effort to explore.

Early growth response (EGR) gene family encompasses four family members: *EGR1*, *EGR2*, *EGR3*, and *EGR4*, locating on 5q31, 10q21, 8p21, and 2p13, respectively (Go et al., 2019). They are transcription factors that contain three highly conserved zinc finger domains in the C-terminus, which recognize GC-rich consensus sequences of the promoters of multiple target genes. Besides, four EGR proteins also contain a transcriptional activation domain in N-terminus (Bhattacharyya et al., 2013).

EGR1 acts as an anti-oncogene engaging in multiple cancer processes, including cancer cell proliferation, apoptosis, and migration and even affects tumor microenvironment (Li et al., 2019; Tang et al., 2019). EGR1 decreased cell growth through downregulating EPO-R transcription under hypoxia in non-small cell lung carcinoma (Su et al., 2019). EGR2 induces cell apoptosis *via* upregulating BNIP3L and BAK in a PETN-dependent manner (Unoki and Nakamura, 2003). EGR3 is also defined as a tumor suppressor, which inhibits cell proliferation and induces apoptosis in hepatocellular carcinoma *in vitro* (Li et al., 2012; Miao et al., 2017; Zhang et al., 2017). EGR4 is abundantly expressed in cholangiocarcinoma tissue and the low expression of EGR4 retards cell growth of cholangiocarcinoma (Gong et al., 2020).

Although a crowd of studies elucidate the mechanism of four members of the EGR family for plentiful types of cancers, the landscape of the prognostic value and role of EGR1 are poorly explored in BRCA. Currently, updated public databases based on integrative bioinformatics analysis of the Cancer Genome Atlas (TCGA) have significantly enhanced the efficiency of identification of biomarkers and functional genes in cancerous diseases (Mei et al., 2019, 2020; Dang et al., 2020). Therefore, this study evaluates the transcriptional profiles and potential prognostic value of the EGR family by systematical bioinformatics analysis and provides a novel role of EGRs in the prognostic value of BRCA.

## Materials and Methods

### Oncomine Analysis

The mRNA expression of EGR1--4 of multiple cancers was retrieved from the Oncomine platform^1^ (Rhodes et al., 2004). The expression among different cancers could be presented on the heat map. The color presents mRNA expression of target genes with overexpression (red) or downexpression (blue).

### TCGA Data Acquisition

The RNA-sequencing and clinical information of BRCA patients in TCGA dataset were downloaded from UCSC Xena^2^. The level of gene expression was measured as log_2_(*x*+1)-transformed RSEM-normalized count. A total of 1,104 BRCA patients were included in our research. The relationship between EGR expression and the clinical features were explored.

### Kaplan-Meier Plotter Analysis

The prognostic value of the EGR family members to RFS was analyzed by the Kaplan-Meier plotter (KM plotter)^3^ (Lanczky et al., 2016). The clinical outcome was displayed with hazard ratio (HR), 95% confidence interval (95% CI), and log-rank *P-*value calculated by algorithms set in the KM plotter.

### Protein-Protein Interaction Network Construction and Enrichment Analysis

Protein-protein interaction (PPI) network was been constructed by GeneMANIA^4^ and visualized by Cytoscape 3.7.2 (Warde-Farley et al., 2010). DAVID^5^ is a widely applied gene functional annotation tool (Dennis et al., 2003). In this study, DAVID was applied to perform Gene Ontology (GO) and Kyoto Encyclopedia of Genes and Genomes (KEGG) analyses of EGRs and their cooperators. The human genome (*Homo sapiens*) was set as the background variables.

### TIMER Analysis

Tumor Immune Estimation Resource (TIMER)^6^ is a beneficial tool to detect tumor-infiltrating immune cells (TIICs) *via* using the RNA-seq expression profiles, including B cells, CD4^+^ T cells, CD8^+^ T cells, neutrophils, macrophages, and dendritic cells (Li et al., 2017). The association between immune infiltrates cells and the expression levels of EGR family members was detected through the TIMER platform, which was displayed by the Pearson method.

### MethSurv Analysis

MethSurv^7^ was used to explore the DNA methylation of EGR1–4 in TCGA (Modhukur et al., 2018). The methylation levels and prognostic values of each CpG in EGR1–4 were analyzed. The patients were divided into low and high methylation groups which were split at the best cut-off point.

### Cell Culture

MCF10A mammary epithelial cell line and BRCA cell lines (MDA-MD-231, MCF-7, and SUM1315) were purchased from the Cell Bank of the Chinese Academy of Sciences (Shanghai, China). All cells were grown in Dulbecco’s modified Eagle’s medium (DMEM) (high glucose) (REF 12800-017, Gibco, United States) supplemented with 10% (*V*/*V*) fatal bovine serum (FBS) (catalog no. SH30396.03, HyClone) and 1% penicillin/streptomycin (REF 15070-063, Gibco) in a humidified incubator at 37°C with 5% CO_2_. Cell lines were testified to be mycoplasma negative monthly.

### RNAi and Transient Transfections

For gene knockdown, small interfering RNA (siRNA) duplex specific for EGR1: siRNA-1 (On-Target Plus: 5′-CCAU GGACAACUACCCUAATT-3′ and 5′-UUAGGGUAGUUG UCCAUGGTT-3′; GenePharma, Shanghai, China), siRNA-2 (On-Target Plus: 5′-GCCUAGUGAGCAUGACCAATT-3′ and 5′-UUGGUCAUGCUCACUAGGCTT-3′; GenePharma, Shanghai, China), siRNA-3 (On-Target Plus: 5′-UCCCAGGACAAUUGAAAUUTT-3′ and 5′-AAUUUCAAU UGUCCUGGGATT-3′). All siRNAs were transfected into MCF10Acell using Lipofectamine 2000 Reagent (REF 11668-019, Invitrogen). The cells were switched to fresh medium with 10% FBS without penicillin/streptomycin for 6 h after transfection and cultured for 24–48 h. Knockdown efficiency was evaluated after transfection for 24 h by measuring mRNA and protein levels using qRT-PCR and Western blotting.

### Western Blotting Analysis

MCF10A, MDA-MD-231, MCF-7, and SUM1315 cell lines seeded into 60-mm dishes/24-well (Thermo Fisher Scientific) were washed with PBS and then lysed with 2 × SDS sample buffer. The lysates were harvested, and abundant protein extracts were separated by 10% SDS-PAGE. The following antibodies were used anti-EGR1 (1:1,000 dilution; catalog no. 55117-1-AP, Proteintech) and anti-β-actin (catalog no. AB21181, Bioworld). Protein levels were normalized to β-actin.

### Boyden Chamber Assay

Cell migration was estimated in a modified Boyden chamber (Coster, Corning, NY), in which two chambers were separated by a polycarbonate membrane (8.0-μm pore diameter). The upper chamber membrane was rendered into single cell suspensions (1 × 10^5^ cells) in serum-free DMEM supplied with 5 μg/ml BSA, and the lower chamber was filled with DMEM with 10% FBS. The cells were allowed to migrate for 12 h at 37°C. The medium was then discarded, washed with PBS, and the cells fixed with 4% paraformaldehyde with PBS. The stationary upper cells were dislodged with a cotton-tipped applicator, and the lower chamber membrane was stained with 0.5% crystal violet. The approximate number of cells that crossed over the membrane was counted by a microscope (Olympus Corporation, Tokyo, Japan).

### Immunohistochemistry

This study was approved by the Ethics Committee of Nanjing Medical University. BRCA tumor tissue microarray (TMA) HBre-Duc060CS-01 (30 cancer cases containing tumor and paired paracancerous tissues) was supplied by Outdo Biotech (Shanghai, China). A series of progresses of immunohistochemistry (IHC) were directly conducted on the TMA. The primary antibodies used were anti-EGR1 (1:100 dilutions) for overnight. DAB and hematoxylin counterstain were applied to visualize its expression. The percentage of positively stained cells was scored as 0–4: 0 (<5%), 1 (6–25%), 2 (26–50%), 3 (51–75%), and 4 (>75%). The staining intensity was scored as 0–3: 0 (negative), 1 (weak), 2 (moderate), and 3 (strong). The expression of EGR1 was assessed by immunoreactivity score (IRS) equaling to the percentages of positive cells multiplied with staining intensity. IRS was employed without prior knowledge of clinical response. Immunostained sections were scanned by a microscope (Olympus Corporation, Tokyo, Japan).

### Statistical Analysis

A series of statistical analyses were conducted through the bioinformatics database online. The GraphPad Prism 8.0 was used to analyze the TCGA data. Student’s *t*-test and one-way ANOVA were used for the EGR mRNA expression levels. Scatter plot charts show scatter plots and means ± SEM. Differences were considered significant if *P-*values were less than 0.05 in all circumstances.

## Results

### The mRNA Expression Levels of EGR Family Across Various Cancers

**FIGURE 1:**
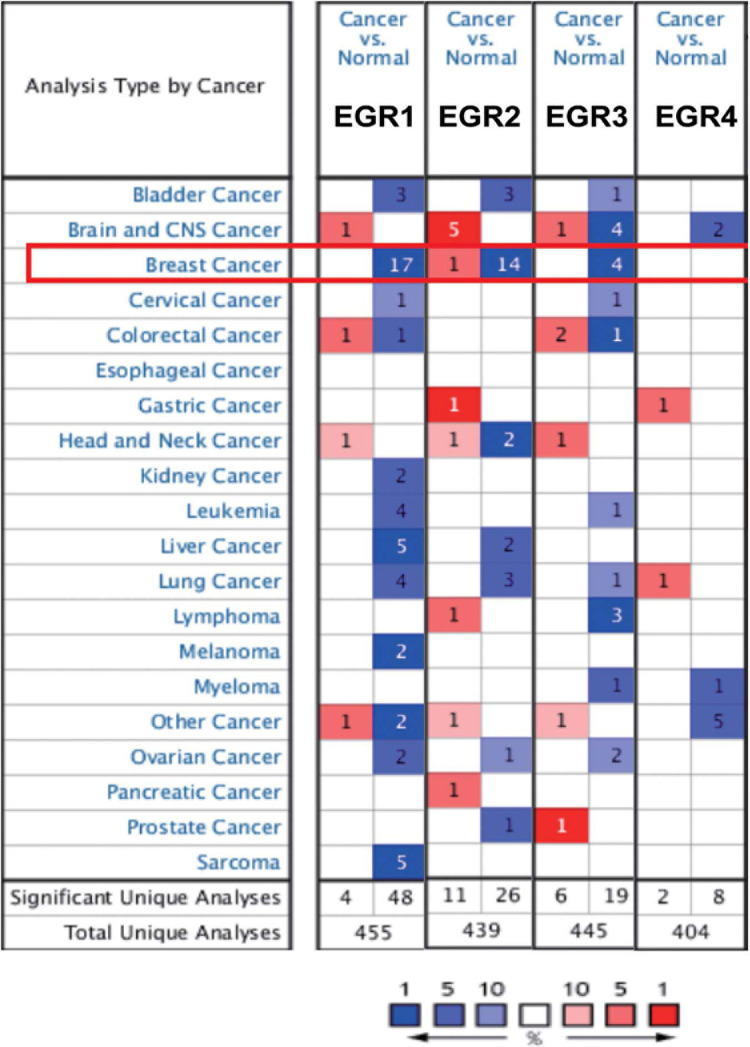
The transcriptional levels of EGRs in different cancers. The differential expressions of EGRs in diverse cancers. The data were derived from Oncomine. Red represented increased expression and blue represented decreased expression. The numbers indicated the amounts of dataset satisfying the threshold in the colored cell.

For the sake of understanding a pan-cancer view of EGRs’ expression, the mRNA expression levels of EGR1–4 on the Oncomine were analyzed. The expressions of EGR1 and EGR3 in 20 different types of human cancers were downregulated compared with that in normal tissues, including BRCA, lung cancer, and ovarian cancer (Figure 1). These results indicated EGR1 and EGR3 might be tumor suppressors. However, the expression of EGR2 was not synchronous in different cancers (Figure 1). Moreover, EGR1, EGR2, and EGR3 remarkably downregulated in BRCA tissues compared with those in normal tissues (Figure 1). The mRNA expression level of EGR4 was absent in BRCA (Figure 1). In total, the mRNA expression levels of EGR1/2/3 were negatively correlated with EGR4 and more studies should be devoted to explore the biological mechanism in various tumors.

### The Transcriptional Levels of EGR Family Members in BRCA

**FIGURE 2:**
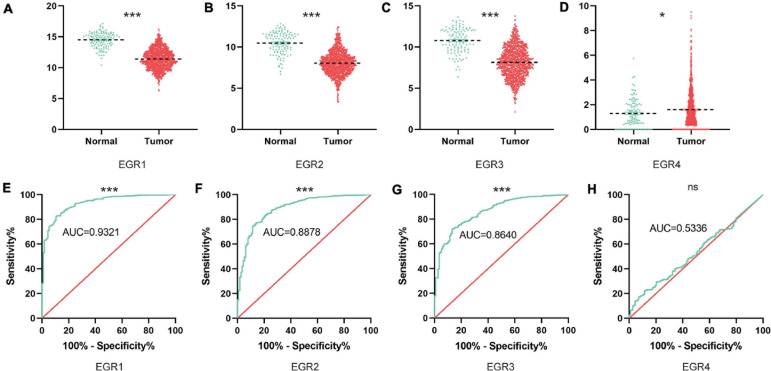
The mRNA levels of EGRs in BRCA tissues based on TCGA. The downregulated expression levels of EGR1/2/3 **(A–C)** and the upregulated expression level of EGR4 **(D)** between normal breast tissues and BRCA were exhibited based on the TCGA website. ***P < 0.001; *P < 0.05. The receiver operating characteristic curves (ROC) of EGRs are shown as well **(E–H)**.

To further investigate the potential value of EGRs in BRCA patients, the different transcriptional levels of EGR1–4 were analyzed based on the TCGA database. From this result, EGR1 (*P* < 0.001), EGR2 (*P* < 0.001), and EGR3 (*P* < 0.001) presented a remarkable downregulation, while EGR4 (*P* < 0.001) was significantly upregulated in BRCA compared with the paracancerous tissues (Figures 2A–D). The receiver operating characteristic (ROC) curves for the expression level of EGR1 (AUC = 0.9321), EGR2 (AUC = 0.8878), and EGR3 (AUC = 0.8640) were meaningful except EGR4 (Figures 2E–H). Thus, EGRs except EGR4 probably had similar molecular roles of BRCA with enhancive coexpression.

### The Association of mRNA Expression of EGRs With Clinical Features

**FIGURE 3:**
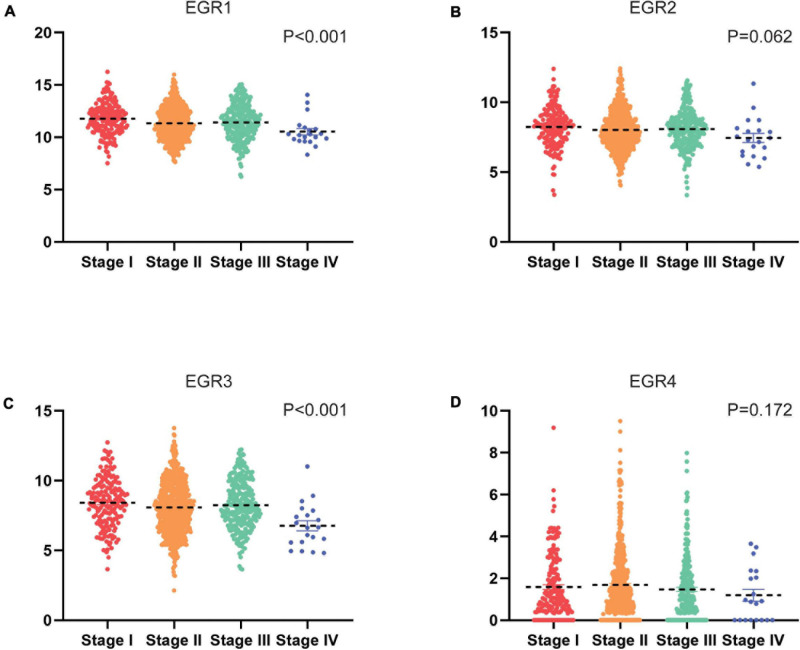
Transcriptional levels of EGRs in different clinical stages. The mRNA expression of EGR1 **(A)**, EGR2 **(B)**, EGR3 **(C)**, and EGR4 **(D)** in clinical stages based on TCGA, including stages I, II, III, IV, and V.

In the low expression of EGR1/2/3 and high expression of EGR4 in BRCA, we wondered whether the expression levels of EGRs might correlate with advanced clinical features of BRCA patients. We evaluated the correlation of transcriptional levels of EGRs and clinical characteristics of BRCA patients, including pathological stages and ER/PR/HER2 status. The mRNA expression of EGR1 (*P* < 0.001), EGR3 (*P* < 0.001) displayed stage-specific expression. The patients with advanced pathological stages expressed lower EGR1/3 mRNA levels. Among four stages, the lowest levels of EGR1/3 were noticed in stage IV (Figures 3A,C). However, the expression levels of EGR2/4 had no obvious correlation with tumor stages (Figures 3B,D).

**FIGURE 4:**
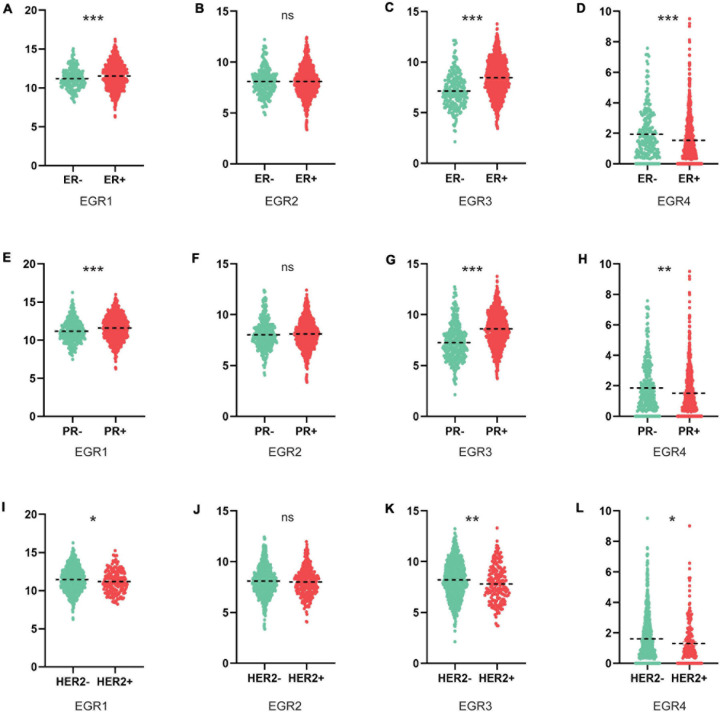
Association between mRNA expression levels of EGRs and ER/PR/HER2 status. The mRNA levels of EGR1/3/4 were significantly associated with ER/PR/HER2 status, while EGR2 expression had uncorrelated to ER/PR/HER2 status **(A–L)**. *P < 0.05; **P < 0.01; ***P < 0.001.

We further compared the transcriptional levels of EGRs in BRCA tissues with different ER/PR/HER2 status. We found EGR1 mRNA expression was increased in the ER^+^/PR^+^ BRCA tissues, which was opposite to HER2^+^ tissues with decreased expression level of EGR1 (Figures 4A,E,I). The upregulated EGR3 was significantly associated to ER^+^/PR^+^ status, but the downregulated EGR3 was significantly correlated to HER2^+^ status (Figures 4C,G,K). For EGR4, the relationship of mRNA level was significantly downregulated in BRCA tissues with ER^+^/PR^+^/HER2^+^ status (Figures 4D,H,L). However, the expression level of EGR2 was unrelated to ER/PR/HER2 status (Figures 4B,F,J). These results implied that the transcriptional levels of EGRs were immensely related to clinical characteristics in BRCA and could be identified as potential biomarkers for the poor differentiation and metastasis status.

### The Prognostic Values of EGRs in BRCA

**FIGURE 5:**
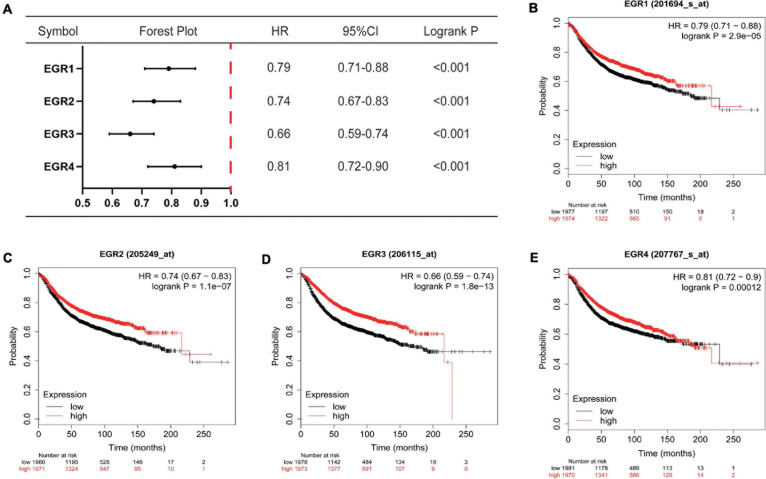
The prognostic values of EGRs for RFS. **(A)** Forest map of prognostic values of EGRs in BRCA patients. **(B**–**E)** Survival curves of EGR1, EGR2, EGR3, and EGR4 were plotted for RFS of patients in BRCA through KM Plotter platform.

The prognostic values in RFS of EGRs were assessed through KM plotter. The high mRNA expression of EGR1 (HR = 0.79, 95% CI: 0.71–0.88, *P* < 0.001), EGR2 (HR = 0.74, 95% CI: 0.67–0.83, *P* < 0.001), EGR3 (HR = 0.66, 95% CI: 0.59–0.74, *P* < 0.001), and EGR4 (HR = 0.81, 95% CI: 0.72–0.90, *P* < 0.001) was correlated with favorable RFS of RBCA patients (Figure 5). These results suggested EGR1–4 were associated with RFS, which could be considered prospective biomarkers to predict survival times of BRCA patients.

### PPI and Enrichment Analysis of EGR Family

**FIGURE 6:**
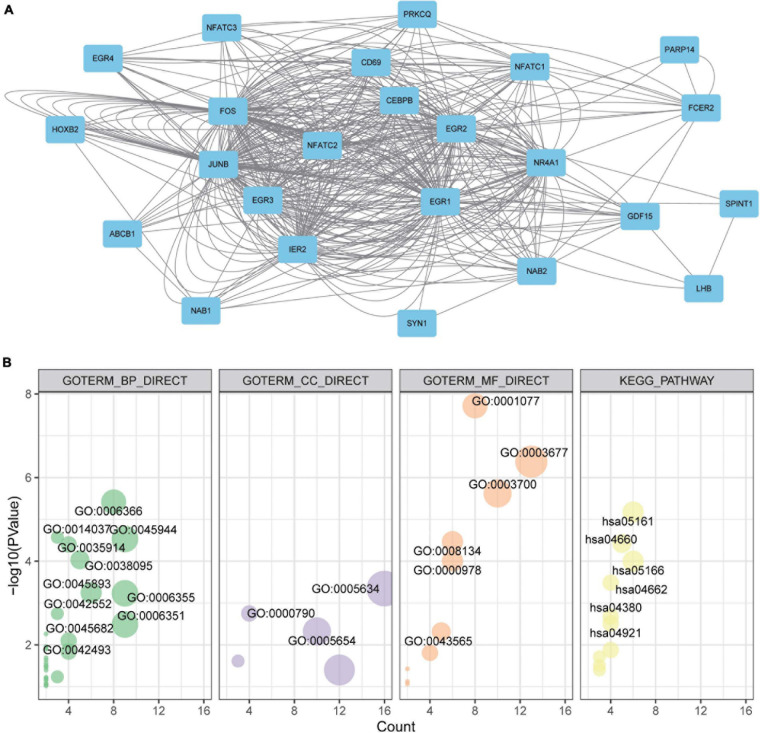
Protein-protein interaction (PPI), Gene Ontology (GO) enrichment, and Kyoto Encyclopedia of Genes and Genomes (KEGG) pathway analyses of EGRs. **(A)** The PPI networks of EGR family. Node, proteins; line, predicted interactions. **(B)** GO enrichment and KEGG pathway analyses of EGRs and their interacted protein *via* DAVID. GO enrichment included cellular component, biological process, and molecular function.

Under the knowledge of the potential values of EGRs for BRCA patients, a mutual PPI network of EGRs was constructed *via* GeneMANIA (Figure 6A). To seek their functions, EGRs and their relevant genes were submitted for GO and KEGG analyses. The results showed that EGR-related genes mainly participated in transcription from RNA polymerase II promoter, positive regulation of transcription from RNA polymerase II promoter, regulation of transcription, and located in nucleus, nuclear chromatin, nucleoplasm. Also, they mediated transcriptional activator activity, DNA binding, transcription factor activity, and enriched in hepatitis B, T cell receptor signaling pathway, B cell receptor signaling pathway, and MAPK signaling pathway (Figure 6B). These data supplied the essential foundation for EGRs participating in the exploration of pathological mechanism and biological role of BRCA.

### The Correlation Between TIICs and EGR Family Members

**FIGURE 7:**
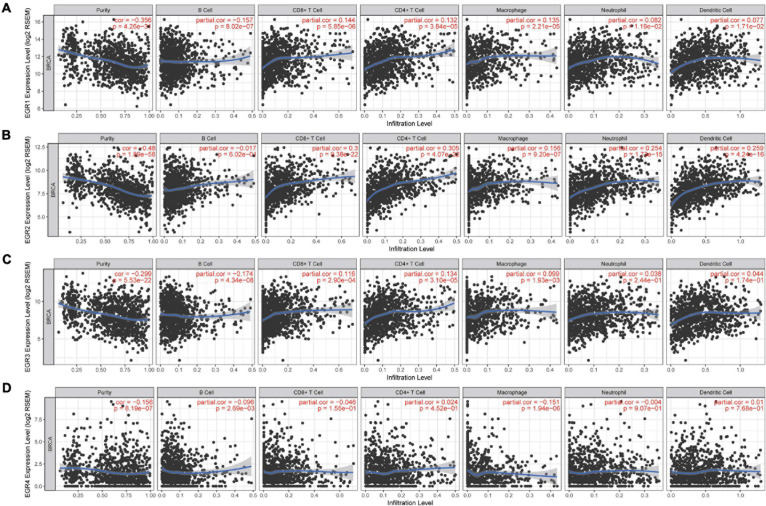
Correlation of TIICs and EGRs. Tumor purity was exhibited at the left panel. The relationship of EGR members and tumor-infiltrating immune cells (B cells, CD4^+^ T cells, CD8^+^ T cells, neutrophils, macrophages, and dendritic cells) is shown, respectively **(A–D)**.

With the development of immunotherapy, the association between immunological characteristics and tumor progression is increasingly focused. Therefore, we further studied the correlation between TIICs and EGR1–4 through the TIMER platform. The expression of EGRs against tumor purity was shown a negative association. Moreover, immune-infiltrated CD8^+^ T cells (cor = 0.3, *P* = 9.38e−22) and CD4^+^ T cells (cor = 0.305, *P* = 4.07e−22) were associated with the expression of EGR2. The correlation of CD4^+^ T cell and EGR2 expression was the highest (Figures 7A–D).

### Prognostic Values of EGR1–4 DNA Methylation in MethSurv

**FIGURE 8:**
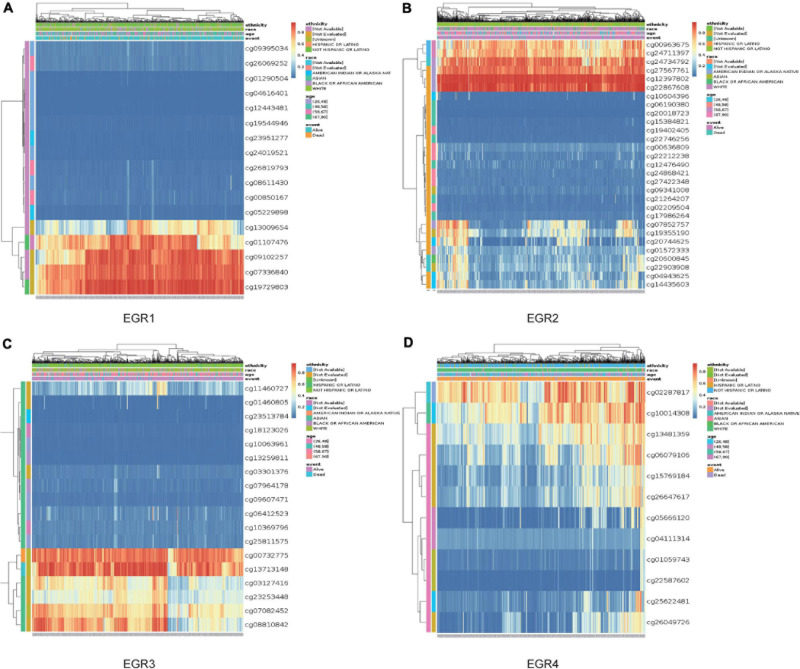
DNA methylation of EGRs in MethSurv. The DNA methylation clustered expression of EGR1 **(A)**, EGR2 **(B)**, EGR3 **(C)**, and EGR4 **(D)**. Red to blue: high to low levels. Annotations were applied to describe the ethnicity, race, age, and event.

MethSurv was employed to detect the DNA methylation levels of EGR1–4 and the prognostic value of each CpG in TCGA (Table 1). Eight CpGs of EGR1, seven CpGs of EGR2, three CpGs of EGR3, and two CpGs of EGR4 were relevant to meaningful prognostic impact. Cg19729803 of EGR1, cg12397802 of EGR2, cg13713148 of EGR3, and cg02287817 of EGR4 revealed the highest DNA methylation levels (Figures 8A–D). These CpG sites of EGRs were largely advantageous for the exploration of the biological mechanism of BRCA.

**TABLE 1:** Prognostic values of EGR family expression and methylation in BRCA patients with different CpG sites.

**Symbol**	**Genomic region**	**Island**	**CpG site**	**HR**	***P*-value**
EGR1	Body	Island	cg07336840	0.60	**0.009**
EGR1	Body	Island	cg09102257	0.65	**0.035**
EGR1	TSS1500	Island	cg26069252	1.39	0.100
EGR1	TSS1500	Island	cg26819793	1.57	0.064
EGR1	TSS200	Island	cg12443481	0.53	**0.009**
EGR1	TSS200	Island	cg19544946	1.21	0.410
EGR1	5′UTR; 1stExon	Island	cg05229898	0.62	**0.019**
EGR1	TSS200	Island	cg24019521	1.70	**0.019**
EGR1	5′UTR; 1stExon	Island	cg23951277	0.62	**0.028**
EGR1	TSS1500	Island	cg00850167	1.52	**0.039**
EGR1	Body	Island	cg13009654	1.44	0.082
EGR1	TSS200	Island	cg01290504	0.83	0.370
EGR1	TSS200	Island	cg01290504	0.59	**0.002**
EGR1	TSS200	Island	cg08611430	1.45	0.085
EGR1	TSS200	Island	cg09395034	0.84	0.410
EGR1	3′UTR	S-Shore	cg01107476	1.18	0.410
EGR1	Body	S-Shore	cg19729803	0.78	0.220
EGR2	Body	Island	cg27567761	0.72	0.130
EGR2	5′UTR	Island	cg10604396	0.48	0.850
EGR2	5′UTR	Island	cg04943625	0.76	0.180
EGR2	5′UTR	Island	cg20744625	1.15	0.520
EGR2	5′UTR	Island	cg14435603	2.02	**0.002**
EGR2	TSS200; 5′UTR	Island	cg06190380	1.39	0.130
EGR2	TSS200; 5′UTR	Island	cg15384821	0.72	0.170
EGR2	TSS200; 5′UTR	Island	cg12476490	0.68	0.073
EGR2	TSS200; 5′UTR	Island	cg17986264	1.56	0.060
EGR2	TSS200; 5′UTR	Island	cg20018723	1.22	0.320
EGR2	TSS200; 5′UTR	Island	cg22746256	1.88	**0.002**
EGR2	TSS200; 5′UTR	Island	cg21264207	1.83	**0.003**
EGR2	5′UTR; 1stExon	Island	cg09341008	1.25	0.290
EGR2	5′UTR; TSS1500	Island	cg02209504	0.69	0.062
EGR2	5′UTR; 1stExon	Island	cg19355190	0.60	**0.013**
EGR2	5′UTR; TSS1500	Island	cg19402405	1.20	0.420
EGR2	5′UTR; TSS1500	Island	cg22212238	1.91	**0.005**
EGR2	5′UTR; TSS1500	Island	cg27422348	1.11	0.640
EGR2	5′UTR; TSS1500	Island	cg24868421	0.95	0.790
EGR2	Body	Island	cg01572333	1.58	**0.022**
EGR2	Body	Island	cg07852757	1.13	0.550
EGR2	Body	Island	cg22867608	1.55	**0.032**
EGR2	Body	Island	cg12397802	0.68	0.064
EGR2	3′UTR	N-Shore	cg00963675	1.23	0.340
EGR2	3′UTR	N-Shore	cg24711397	1.26	0.340
EGR2	5′UTR; 1stExon	N-Shore	cg22903908	0.69	0.072
EGR2	TSS200	S-Shore	cg20600845	0.79	0.310
EGR2	TSS1500	S-Shore	cg24734792	1.16	0.500
EGR3	TSS200	Island	cg13259811	0.85	0.470
EGR3	1stExon	Island	cg18123826	0.73	0.110
EGR3	1stExon	Island	cg10369796	1.08	0.690
EGR3	1stExon; 5′UTR	Island	cg23513784	0.62	**0.016**
EGR3	Body	Island	cg03127416	0.78	0.290
EGR3	Body	Island	cg01460805	0.75	0.210
EGR3	Body	Island	cg03301376	1.42	0.081
EGR3	Body	Island	cg08810842	1.45	0.090
EGR3	TSS1500	Island	cg10063961	0.65	**0.040**
EGR3	Body	Island	cg11460727	0.88	0.500
EGR3	Body	Island	cg23253448	0.37	0.120
EGR3	TSS1500	Island	cg25811575	1.48	0.064
EGR3	TSS1500	Island	cg06412523	1.19	0.430
EGR3	3′UTR	Island	cg07082452	1.17	0.510
EGR3	TSS1500	Island	cg09607471	0.62	**0.025**
EGR3	TSS200	Island	cg07964178	0.68	0.056
EGR3	3′UTR	N-Shore	cg00732775	1.15	0.490
EGR3	3′UTR	N-Shelf	cg13713148	1.51	0.063
EGR4	1stExon	Island	cg04111314	0.76	0.160
EGR4	1stExon	Island	cg05666120	1.15	0.540
EGR4	5′UTR; 1stExon	Island	cg01059743	1.61	**0.042**
EGR4	5′UTR; 1stExon	Island	cg22587602	0.82	0.360
EGR4	Body	Island	cg06079106	0.78	0.200
EGR4	Body	Island	cg15769184	1.16	0.460
EGR4	Body	Island	cg13481359	1.49	0.095
EGR4	Body	Island	cg26049726	1.21	0.410
EGR4	Body	Island	cg26647617	1.13	0.540
EGR4	3′UTR	Island	cg25622481	1.16	0.460
EGR4	TSS1500	S-Shore	cg02287817	0.66	0.072
EGR4	3′UTR	N-Shore	cg10014308	0.59	**0.009**

*Bold fonts indicate significant differences.*

### The High Expression of EGR1 in BRCA Paracancerous Tissues and Its Migration Resistant Role in Mammary Epithelial Cell

**FIGURE 9:**
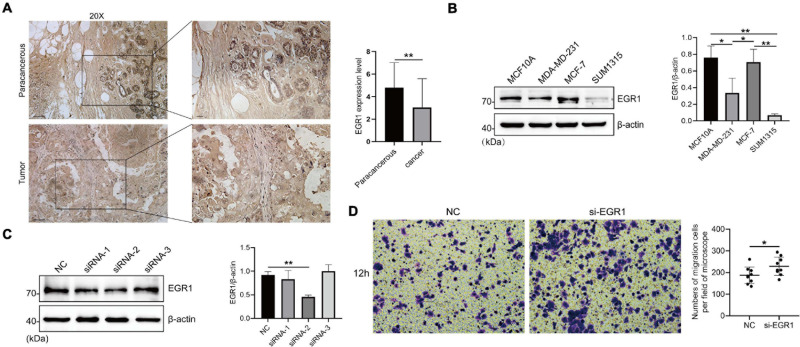
The high expression of EGR1 in BRCA paracancerous tissues and its migration-resistant role in mammary epithelial cells. **(A)** IHC staining of EGR1 in BRCA and paracancerous tissues. **(B)** The expression level of EGR1 in MCF10A mammary epithelial cell and BRCA cells (MDA-MB-231, MCF-7, and SUM1315) were examined by Western blotting. **(C)** The transfection efficiency of siRNA-1, siRNA-2, and siRNA-3 was measured by Western blotting. **(D)** The migration of MCF10A mammary epithelial cell after the transfection of si-EGR1 was measured by Boyden chamber assays. The numbers of migration cells per field of microscope were counted. *P < 0.05, **P < 0.01.

Based on numerous bioinformatics analyses of EGRs, we found that EGR1–4 showed distinct transcriptional expression level between BRCA and paracancerous samples and presented significant prognostic value in RFS. Thus, we examined the protein expression and the effect of EGR1 on cell migration by immunostaining, western blotting, and Boyden chamber assay. IHC staining showed that the EGR1 was remarkably downregulated expression in BRCA compared with that in paracancerous tissues, which corresponded with the findings from bioinformatics analysis (Figure 9A). Similarly, the expression level of EGR1 was significantly decreased in MDA-MD-231 and SUM1315 cells compared with that in MCF10A mammary epithelial cell, except MCF-7 (Figure 9B).

Next, we measured the knockdown efficiency after the transfection of siRNA-1, siRNA-2, and siRNA-3 targeting EGR1. The knockdown efficiency of siRNA-2 targeting EGR1 was the highest (Figure 9C). Boyden chamber assay exhibited that MCF10A had an increased migration capacity after EGR1 silence (Figure 9D). To sum up, these findings preliminarily suggested an antioncogene role of EGR1 in BRCA.

## Discussion

Based on online databases, we discovered EGR1/2/3 expression levels were significantly downregulated, while EGR4 was upregulated in BRCA tissues. The prognostic values of EGR1–4 showed a positive relationship with better RFS of BRCA patients. Although accumulating evidences confirm EGRs regulate the initiation and/or development of multiple cancers, the expression profile and prognostic value of EGR1–4 and the role of EGR1 in BRCA remain unclear (Suzuki et al., 2007; Li et al., 2019). According to experiment validations, our investigations found that EGR1 protein was highly expressed in paracancerous tissue and resisted the migration of MCF10A cells. It is the first time to systemically and comprehensively analyze the expression levels, potential prognosis, TIICs status, and DNA methylation level of EGR1–4 in BRCA by bioinformatics methods.

EGR1, considered a tumor suppressor, is negatively associated with poor prognosis and early recurrence. Yang Y. et al. (2019) reported overexpressed EGR1 repressed cell apoptosis and promoted cell proliferation by interacting with DNMT3L to inhibit the miR-195-AKT3 pathway in gastric cancer. In this study, EGR1 was expressed at a remarkably lower level in BRCA tissues than that in paracancerous tissues. Upregulated EGR1 mRNA expression was notably correlated with ER^+^/PR^+^ status, and the downregulation of EGR1 was associated with HER2^+^ status. The high expression of EGR1 exhibited a correlation with fine RFS. Crawford et al. (2019) found the expression of EGR1 was reduced in BRCA, which was in agreement with our results. Besides, active EGR1 elevated PAC1 expression with excessive oxygen species, ultimately causing the chromatin remodeling mechanism of effector T cells (Dan et al., 2020). Analogously, we found immune-infiltrated cells were related to the mRNA expression of EGR1 from the TIMER platform, such as B cell, CD8^+^ T cell, and macrophage cell.

Owing to the significant difference of the transcriptional level, clinical characteristics, prognostic value, PPI, TIICs, and DNA methylation of EGR1, we further explored the protein expression of EGR1 by western blotting. Also, the role of EGR1 in cell migration was determined by Boyden chamber assay. Overexpressed miR-125b-2-3p notably increased lymphatic invasion and distant migration by targeting EGR1 in clear cell renal cell carcinoma (Meng et al., 2020). Similarly, our result showed that cell migration of human mammary epithelial cell MCF10A was increased when EGR1 was silenced.

In our study, EGR2 expression was decreased and high expression of EGR2 was related to favorable RFS, indicating its prognostic value in BRCA. However, EGR2 had no significant difference of PR^–/+^/ER^–/+^/HER2^–/+^ status, which might need further research. EGR3 is frequently declined in hepatocellular carcinoma tissues, retarded cell proliferation, and induced apoptosis *in vitro* (Zhang et al., 2017). The microarray data revealed a decreased expression of EGR3 especially acted as a potential candidate gene for the diagnosis and prognosis of cutaneous squamous cell carcinoma (Wei et al., 2018). Interestingly, our results displayed the upregulation of EGR3 was largely correlated with good RFS in BRCA.

EGR2 and EGR3 play important roles in adjusting the transition between proliferation and differentiation of effector CD4^+^ and CD8^+^ T cells (Miao et al., 2017; Yang R. et al., 2019). In our report, EGR2 was strongly related to CD8^+^ T cell, CD4^+^ T cell, macrophage cell, neutrophil cell, and dendritic cell. EGR3 presented a conspicuous association with immune infiltrate cells as well, like B cell, CD8^+^ T cell, CD4^+^ T cell, and macrophage cell. Gong et al. (2020) found EGR4 facilitated tumor cell growth with high expression in cholangiocarcinoma. Surprisingly, EGR4 was highly expressed and had a significantly negative association with ER^+^/PR^+^/HER2^+^ status. In BRCA, EGR4 expression presented a positive correlation with better RFS of BRCA patients. The biological function and molecular processes of EGR4 in cancers was still rarely discovered.

Up to now, the study of methylation of EGRs remains limited. In our analysis, the DNA methylation heat maps were clearly shown in all CpG islands. Moreover, DNA methylation levels in several EGR CpG islands displayed significant association with prognosis of BRCA patients.

## Conclusion

We systematically analyzed the transcriptional levels and prognostic values of EGRs in BRCA *via* public databases. Our finding reveals that EGRs are possible to be novel prognostic biomarkers for BRCA patients. Besides, EGR1/2/3 are promising prognostic biomarkers for predicting RFS of BRCA patients. This study provides a comprehensive insight into the characteristic investigation of the EGR family and the role of EGRs in the prognostic value of BRCA.

## Data Availability Statement

The original contributions presented in the study are included in the article/supplementary material, further inquiries can be directed to the corresponding author/s.

## Ethics Statement

The studies involving human participants were reviewed and approved by the Ethics Committee of Nanjing Medical University. The patients/participants provided their written informed consent to participate in this study.

## Author Contributions

LH, FH, XY, BX, and YL analyzed the data and wrote the manuscript. YZhu, YZha, and LH designed the study and performed data. LH and YZhu prepared the figures and tables. All authors read and approved the final manuscript.

## Conflict of Interest

The authors declare that the research was conducted in the absence of any commercial or financial relationships that could be construed as a potential conflict of interest.

## Footnotes

1 https://www.oncomine.org/

2 https://xena.ucsc.edu/

3 http://www.kmplot.com/

4 https://genemania.org/

5 https://david.ncifcrf.gov/

6 https://cistrome.shinyapps.io/timer/

7 https://biit.cs.ut.ee/methsurv/

## Abbreviations

BRCA: breast cancer
RFS: release-free survival
EGR: early growth response gene
TCGA: The Cancer Genome Atlas
KM plotter: Kaplan-Meier plotter
HR: hazard ratio
95% CI: 95% confidence interval
PPI: protein-protein interaction
GO: Gene Ontology
KEGG: Kyoto Encyclopedia of Genes and Genomes
TIMER: Tumor Immune Estimation Resource
TIICs: tumor-infiltrating immune cells
ROC: the receiver operating characteristic.
